# 

**Published:** 1869-04

**Authors:** 


### Books and Pamphlets Received.

A Practical Treatise on the Diseases of Women. By T. Gaillard Thomas, M. D., Professor of Obstetrics and the Diseases of Women and Children in the College of Physicians and Surgeons, New York; Physician to Bellevue Hospital; Consulting Physician to the State Woman's Hospital; late President of the New York Obstetrical Society; Member of the New York Academy of Medicine, of the County Medical Society, etc., etc., with two hundred and twenty-five illustrations. Second edition, revised and improved. Philadelphia: Henry C. Lea, 1869. Received through and for sale by Theo. Butler & Son, Buffalo.

The Intermarriage of Relations. By Nathan Allen, M. D.

On the Treatment of Paralysis by Electrization, with an Explanation of a New Galvanic Apparatus; being a paper read before the New York Academy of Medicine, November 19, 1868.

The Probe; an Inquiry into the use of Stimulants and Narcotics, the Social Evils resulting therefrom, and Methods of Reform and Cure. By Joseph Parrish, M. D.

Address delivered to the Medical Class at the University of Michigan, March 31, 1869. By E. O. Haven, President of the University.

Twenty-sixth Annual Report of the Managers of the State Lunatic Asylum for the year 1868.

Twentieth Annual Announcement of the Woman's Medical College of Pennsylvania, 1869-70.

